# *Neisseria gonorrhoeae* Induces a Tolerogenic Phenotype in Macrophages to Modulate Host Immunity

**DOI:** 10.1155/2013/127017

**Published:** 2013-09-24

**Authors:** Alejandro Escobar, Enzo Candia, Sebastian Reyes-Cerpa, Bélgica Villegas-Valdes, Tanya Neira, Mercedes Lopez, Kevin Maisey, Fabián Tempio, Miguel Ríos, Claudio Acuña-Castillo, Mónica Imarai

**Affiliations:** ^1^Instituto de Investigación en Ciencias Odontológicas, Facultad de Odontología, Universidad de Chile, 8380492 Santiago, Chile; ^2^Laboratorio de Inmunología, Departamento de Biología, Facultad de Química y Biología, Universidad de Santiago de Chile, Santiago, Chile; ^3^Programa Disciplinario de Inmunología, Instituto de Ciencias Biomédicas, Facultad de Medicina, Universidad de Chile, 8380453 Santiago, Chile

## Abstract

*Neisseria gonorrhoeae* is the etiological agent of gonorrhoea, which is a sexually transmitted disease widespread throughout the world. *N. gonorrhoeae* does not improve immune response in patients with reinfection, suggesting that gonococcus displays several mechanisms to evade immune response and survive in the host. *N. gonorrhoeae* is able to suppress the protective immune response at different levels, such as B and T lymphocytes and dendritic cells. In this study, we determined whether *N. gonorrhoeae* directly conditions the phenotype of RAW 264.7 murine macrophage cell line and its response. We established that gonococcus was effectively phagocytosed by the RAW 264.7 cells and upregulates production of immunoregulatory cytokines (IL-10 and TGF-**β**1) but not the production of proinflammatory cytokine TNF-**α**, indicating that gonococcus induces a shift towards anti-inflammatory cytokine production. Moreover, *N. gonorrhoeae* did not induce significant upregulation of costimulatory CD86 and MHC class II molecules. We also showed that *N. gonorrhoeae* infected macrophage cell line fails to elicit proliferative CD4+ response. This implies that macrophage that can phagocytose gonococcus do not display proper antigen-presenting functions. These results indicate that *N. gonorrhoeae* induces a tolerogenic phenotype in antigen-presenting cells, which seems to be one of the mechanisms to induce evasion of immune response.

## 1. Introduction

The gram-negative diplococcus *Neisseria gonorrhoeae* is the causal agent of gonorrhoea, one of the two most common sexually transmitted diseases [1]. Infection susceptibility and colonization mechanisms of gonococcus have been studied using different models that have determined that gonococcal membrane components, such as Pili, Opa, and lipooligosaccharide (LOS), are highly relevant to infection [1]. Infection by gonococcus is associated with several clinical manifestations, such as cervicitis and urethritis, as well as pelvic inflammatory disease, ectopic pregnancy, chronic pelvic pain, and infertility. Strikingly, infections frequently occur without any clinical manifestations that is, more than half of infected women and a significant percentage of infected men never develop symptoms [2–4]. In women, this is also related to persistency and infection to the upper genital tract. In regard to immune response, it has been determined that humoral immunity against gonococcus is highly limited [5, 6] and T cells undergo a transient reduction during infection [7]. Clinical data also indicates that previous infections with *N. gonorrhoeae* do not improve immune response in patients with reinfection, thus suggesting that immunological memory is not induced by gonococcus [5].

The ineffective immune response against gonococcus is multifactorial. It has been hypothesized that it could be the sum of different mechanisms, one of which could be related to genital tissue properties, such as inmune privileged site in the female tract [8, 9], while the other could involve evasion mechanisms intrinsically developed by the bacteria, an idea supported by several lines of evidence; that is, it has been demonstrated that the bacterium undergoes phase and antigenic variations [10] and shows epitope mimicry [11, 12] and phagosome subversion [13] to overcome immune defense. Moreover, gonococci seem to directly interfere with the onset of the adaptive immune response as Opa proteins can inhibit CD4+ T-cell proliferation [14] and the bacteria induce IL-10 production, an important regulatory cytokine involved in the differentiation of type 1 T regulatory cells (Tr1) [15, 16]. More interestingly, data obtained from the murine model of experimental infection showed an increase of CD4+Foxp3+CD25+ regulatory T lymphocytes (Tregs) in the lymph nodes draining of the genital tract. This increase correlated with an augmenting of TGF-*β*1-positive cells in the uterine stroma of infected animals [17]. Recent studies in the murine model of gonococcal genital tract infection showed that *N. gonorrhoeae* enhances TGF-*β*1 production and thereby promotes Th17-dependent response with the consequent deployment of Th1/Th2 protective response [18].

The current data suggest that *N. gonorrhoeae* is able to suppress the protective immune response at different levels, such as B and T lymphocytes [19, 20]. Recently, Duncan et al. demonstrated that *N. gonorrhoeae* potently inhibits the ability of antigen primed bone-marrow-derived dendritic cells (BMDC) to trigger T-cell proliferation by inducing expression of both immunosuppressive cytokines and tolerance-inducing cell surface protein [21]. However, until now there have been no reports on the potential effects of gonococcus on macrophages. Macrophages are an essential component of innate immunity and play a central role in inflammation and host defence [22]. Cells of the monocyte-macrophage lineage are characterized by considerable diversity and plasticity. In response to various signals, macrophages may undergo classical M1 activation (stimulated by toll-like receptor ligands and IFN-*γ*) or alternative M2 activation (stimulated by IL-4/IL-13) [23]. Recently, studies have considered macrophages as a continuum with a range of overlapping functions in which classically activated and regulatory macrophages [24] can influence immune response.

Here, we demonstrate that gonococcus conditions macrophage cell line phenotype and its functionality, producing a shift towards anti-inflammatory cytokine production, inefficient upregulation in molecules involved in antigen presentation and T-cell activation, and weak allogeneic T-cell stimulatory activity. We think that this macrophage phenotype can favor gonococcus against host defence during infection.

## 2. Materials and Methods

### 2.1. Bacteria and Culture Conditions

The *Neisseria gonorrhoeae* P9-17 strain used in this study was kindly provided by Dr. Myron Christodoulides (University of Southampton, UK) [25]. In particular, P9-17 (Pil^+^  Opab^+^) variant of *N. gonorrhoeae* containing the red-shift mutant GFP (rs-GFP) plasmid was used. Bacterial growth and analysis of colony morphology were handled, as previously described [17]. Briefly, gonococcal variants were taken from frozen stocks, plated in GC agar plates (Difco, Becton Dickinson) containing BBL Isovitalex (Becton Dickinson, Sparks, MD, USA), and cultured at 37°C in 5% CO_2_ for 18 to 20 h to obtain single colonies. Single colonies showing the proper morphology were further grown for subsequent experiments. 

### 2.2. Animals and Cell Cultures

Male or female 6 to 8-week-old C57BL/6 mice were obtained from the USACH Research Facility. Animals were euthanized by cervical dislocation, spleens were removed under sterile conditions, and splenocytes were obtained free of erythrocytes by treatment with ACK lysis buffer. Splenocytes were cultured in RPMI 1640 medium (Invitrogen Corporation, Carlsbad, CA, USA) supplemented with 10% FBS (Biological Industries Ltd., Kibbutz Haemek 25115, Israel), 50 U/mL penicillin-streptomycin, and 2.5 *μ*g/mL amphotericin B (Sigma-Aldrich, St. Louis, MO, USA). Research was conducted in accordance with institutional guidelines and the International Guiding Principles for Biomedical Research Involving Animals of the Society for the Study of Reproduction. RAW 264.7 murine macrophages (American Type Culture Collection, Rockville, MD, USA) were cultured in RPMI-1640 medium (Gibco Invitrogen Co., Carlsbad, CA, USA) with 10% fetal bovine serum (FBS, HyClone Laboratories, Inc., Logan, UT, USA), 50 units/mL penicillin, and 50 *μ*g/mL streptomycin (Gibco Invitrogen Co) at 37°C, in 95% air and 5% CO_2_.

### 2.3. Bacterial Infection

*Neisseria gonorrhoeae* P9-17 (Pil^+^, Opab^+^) or fluorescent variant was suspended in phosphate-buffered saline (PBS) and the number of colony forming units (CFU) was determined by optical density at 600 nm. Macrophages were grown in 24-well plates (or in cover slips) using cell-culture medium lacking antibiotics. Nearly confluent monolayers of cells were challenged with *Neisseria gonorrhoeae* at a multiplicity of infection (MOI) of 10 and incubated for the indicated period of times at 37°C with 5% CO_2_. 

### 2.4. Immunofluorescence Microscopy Analysis

After bacterial challenge, cell monolayers were washed five times with media and then fixed for confocal microscopy in 1% paraformaldehyde in PBS (pH 7.4). In order to visualize APC cells, raw monolayers were immunostained with a phycoerythrin- (PE-) conjugated anti-mouse CD11b antibody (1 : 50 Santa Cruz Biotechnology). Association of GFP-fluorescent bacteria with the immunostained APCs was determined microscopically using confocal microscopy (Axiovert 100 M Microscope, Zeiss). In addition, confocal orthogonal analysis was used to identify internalized bacteria. In this case, *z*-plane slices were obtained and orthogonal views and three-dimensional images were generated from isolated cells. 

### 2.5. Gentamicin Protection Assay

Assays were performed as described previously [26]. Briefly, to quantify the total number of macrophage-associated gonococci, treated cells were washed 3–5 times to remove nonadherent bacteria and then lysed with 1% saponin (Sigma, St Louis, MO, USA) in PBS for 30 min. To quantify the number of internalized gonococci, 100 *μ*g/mL of gentamicin (US Biological, Swampscott, MA, USA) was added 1 h prior to the preparation of lysates to kill the extracellular adherent bacteria. The lysates were collected, serially diluted, and aliquots were seeded onto supplemented GC agar plates. After 24 h incubation at 37°C and 5% CO_2_, colony-forming units were counted.

### 2.6. Cytokine Detection

Supernatants were collected after 24 h incubation of macrophages with the bacteria. In each case, stimulation of APCs with LPS from *E. coli* (serotype 055:B5; Sigma) was used at 1 *μ*g/mL as positive control. An enzyme-linked immunosorbent assay was performed to measure cytokines. Briefly, plates were coated with 100 *μ*L/well of the captured antibody and incubated at 4°C overnight. The captured antibodies were anti-IL-10 from clone JES5-16E3 (e-bioscience, CA, USA) and anti-TNF-*α* from clone 1F3F3D4 (e-bioscience). After washing and blocking, samples and standard cytokines were added and plates were incubated for 2 h. Then, plates were washed and 100 *μ*L of the polyclonal antibodies was added. After 1.5 h incubation, plate contents were eliminated, wells were washed several times and biotin-conjugated anti-IL-10 antibody clone JES5-2A5 (e-bioscience) or biotin-conjugated anti-TNF-*α* antibody clone XT3/XT22 (e-bioscience) were added. After 1.5 h incubation, streptavidin-HRP (1 : 5000) was added and again incubated 30 min at 37°C. Detection was performed using Luminol for IL-10 assays or ABTS for TNF*α* assays. Plates were read at 405 nm. Assays were performed in triplicate. 

### 2.7. Immunofluorescence Staining

For TGF-*β*1 detection, cells were collected and 5 × 10^5^ cells were then incubated with an anti-TGF-*β*1 polyclonal antibody (1 : 100, Santa Cruz Biotechnology) for 1 h at 4°C. Cells were washed several times with PBS containing 2% FCS and then incubated with a PE-conjugated polyclonal anti-rabbit IgG (1 : 150; Sigma Chemicals) for 1 h at 4°C. Isotypic controls were routinely included in all experiments. RAW cells were stained with anti-CD11b PE-conjugated (1 : 50; Santa Cruz Biotechnology) for 1 h at room temperature, anti-MHC class II FITC-conjugated (Anti I-A^d^, 1 : 300; Becton Dickinson) for 1 h at room temperature and also with anti-CD86 FITC-conjugated (1 : 200; Becton Dickinson) for 1 h at room temperature. Cells were routinely washed with PBS containing 2% FCS and centrifuged at 600 ×g for 8 min and then resuspended in PBS containing 2% FCS. Analysis was performed by Cyflogic analysis software package Version 1.2.1 (http://www.cyflogic.com/).

### 2.8. Alloproliferative Assay

Splenocytes (5 × 10^4^/well) were labeled with CFSE (5 mM per 1 × 10^7^ cells) (Renovar, USA) for 15 min at 37°C. Cells were washed extensively and 2 × 10^5^ cells/well were cultured with 4 × 10^4^ Mitomycin-treated (16 *μ*g/mL) RAW cells in round-bottomed 96-well plates in cultured in RPMI-1640 medium (Gibco Invitrogen Co, Carlsbad, CA) with 10% fetal bovine serum (FBS, HyClone Laboratories, Inc., Logan, UT), 50 units/mL penicillin and 50 *μ*g/mL streptomycin (Gibco Invitrogen Co) at 37°C, in 95% air and 5% CO_2_ for 5 days. Proliferation analysis was performed Analysis was performed by Cyflogic analysis software package Version 1.2.1 (http://www.cyflogic.com/).

### 2.9. Data Analysis

Data are presented as mean ± standard error (SE). All data were analyzed using GraphPad software. The Kruskal-Wallis test and subsequently Dunn's Multiple Comparison Test were used to evaluate differences among the groups. Differences were considered statistically significant at *P* < 0.05.

## 3. Results

### 3.1. Induction of Cytokines upon Infection of Mouse Macrophage Cell Line with *Neisseria gonorrhoeae*

**Figure 1:**
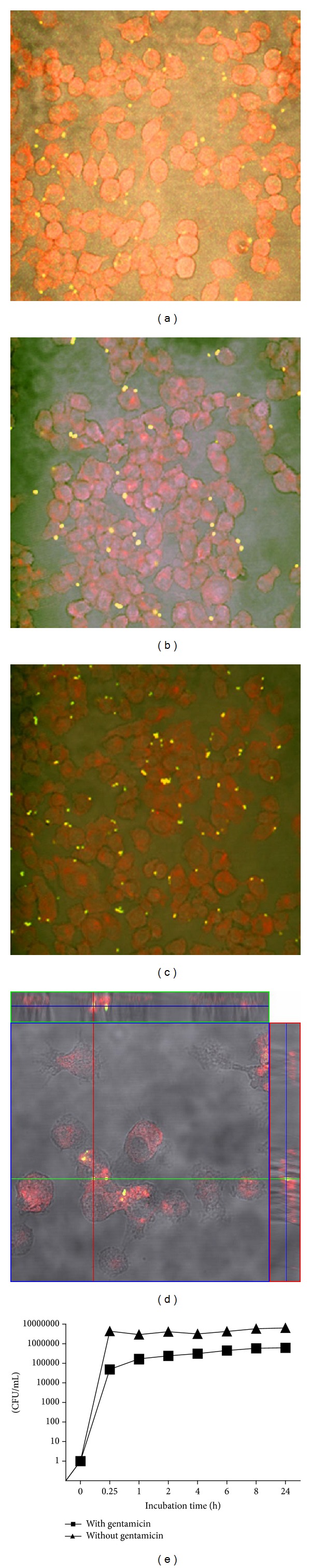
Gonococcus uptake by murine macrophage cell line: fluorescence micrograph of murine macrophage cell line RAW 264.7 incubated with GFP-expressing *Neisseria gonorrhoeae* (green) variant P9-17 for (a) 1 h, (b) 2 h, (c) 3 h, and (d) orthogonal view of the intracellular gonococci (green spots); a midplane *Z*-section of height 1.25 *μ*m is shown. Phase contrast denotes cell boundaries. Nuclei are in red. (e) Gentamicin protection assay for infection of RAW cells with *N. gonorrhoeae* variant P9-17 (*n* = 3).

The phenotype of antigen presenting cells and the cytokine milieu can modulate and define the type of immune response elicited against a pathogen. Therefore, considering that for their part, microorganisms can modulate the phenotype of APC, we wanted to determine the effects that gonococcus might have on the phenotype of macrophages. We first examined interactions occurring between macrophage cell line and gonococci. Therefore, we challenged the RAW 264.7 murine macrophages with variants of *N. gonorrhoeae* fluorescent strain P9-17 (Pil^+^,  Opab^+^). RAW cells and the corresponding bacterial variant were incubated for 1, 2, 3 h at 37°C with 5% CO_2_. Associated bacteria were then evaluated by confocal microscopy. Bacteria were observed to be associated with RAW cells in a time-dependent form (Figures 1(a)–1(c)). Bacterial uptake by macrophages was visualized by sequential cross-sectional images and an orthogonal view of the macrophages (midplane *Z*-section, height 1.25 *μ*m) (Figure 1(d)). In addition, gonococcus internalization was determined by a standard gentamicin protection assay, demonstrating that a significant number of viable bacteria were recovered from the inner compartment of macrophages (Figure 1(e)) and those represent approximately 10% of total macrophage-associated bacteria. Results demonstrated that gonococci were effectively phagocytosed by RAW macrophages.

**Figure 2:**
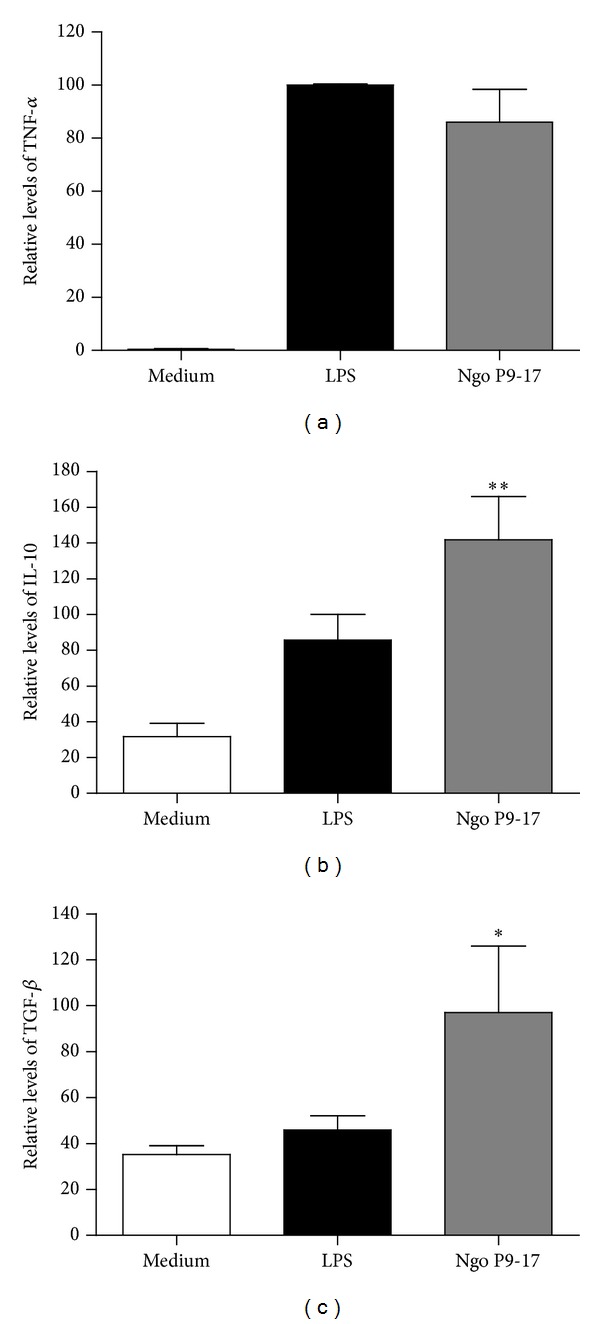
Cytokines induced in murine macrophage cell line infected with *N. gonorrhoeae* P9-17 LPS is the positive control. A statistically significant difference is found when data is compared to the level of cytokines found in the medium from cultures without infection. (a) Secretion of TNF-*α* by RAW cells measured 24 h after challenge. Data represent the levels of cytokine relative to levels induced by LPS. Bars are the mean ± SEM of 6 independent experiments. (b) Secretion of IL-10 after 24 h treatment. *n* = 5  ** indicates that P9-17 induces higher levels of IL-10 than LPS and other treatments *P* < 0.01. (c) TGF-*β*1 levels on the surface of treated macrophages. Values correspond to the mean fluorescence intensity (G-mean) values relative to the levels found in untreated cells. Data are presented as the mean ± SEM of *n* = 7 challenge experiments. * indicates that P9-17 induces higher levels of TGF-*β*1 than LPS and other treatments *P* < 0.05.

Our next aim was to evaluate the type of response induced by the bacteria in comparison to a strong inflammatory stimulus, such as lipopolysaccharide. Thus, macrophage cell line were challenged with P9-17 variant for 24 h. IL-10 and TNF*α* levels in culture supernatants were compared to LPS-elicited response. Data obtained from 6 independent experiments showed that P9-17 induced the production of the pro-inflammatory cytokine TNF*α* (Figure 2(a)) at similar levels that to those of LPS. Otherwise, the results of 5 independent experiments indicate that the anti-inflammatory cytokine IL-10 was induced under all experimental conditions, although in this case P9-17 induced the highest levels compared to the LPS and medium (Figure 2(b)). To better assess the effect of gonococcal infection on immunoregulatory cytokine expression, membrane TGF-*β*1 in RAW cells was measured in 7 independent experiments by flow cytometry. Geomean data from histograms were obtained in each experiment and are shown normalized against LPS treatment. The results demonstrate that TGF-*β*1 was highly induced in response to gonococcal variant P9-17 (Figure 2(c)). The ratio of TNF*α* to IL-10 and to TGF-*β* changes was 1.9 and 3.8, respectively. In summary, when RAW 264.7 murine macrophages were infected, *Neisseria gonorrhoeae* was able to upregulate production of the immunoregulatory cytokines IL-10 and TGF-*β*1.

### 3.2. Gonococcus Affects Activation of Mouse Macrophage Cell Line and Its Proliferative Induction Capacity

**Figure 3:**
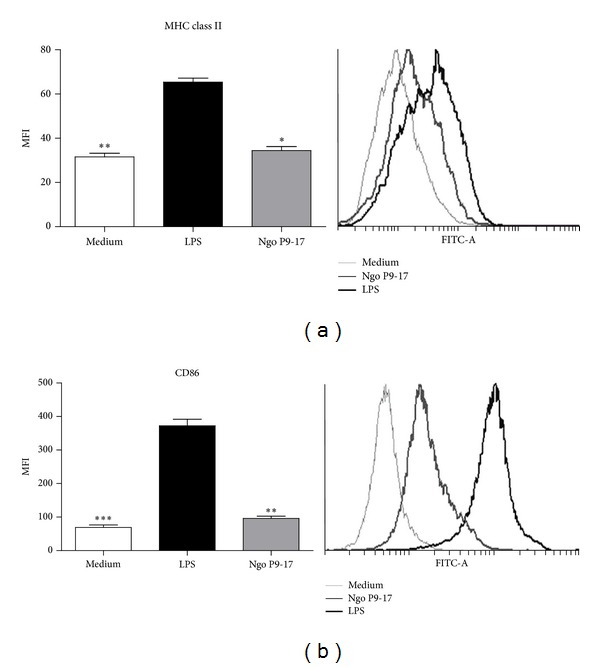
Quantification of MHC II and CD86 expression on the surface of gonococcus-infected RAW cells using flow cytometry. Macrophages were stained with anti-CD11b specific antibodies. (a) MHC class II in RAW cells, *n* = 6. (b) CD86 in RAW cells, *n* = 8. Bars represent mean ± SEM of independent experiments. Representative histogram plot are shown in the right panel. * indicates that P9-17 induced lower levels of MHC II compared to levels induced by LPS, *P* < 0.05; ** indicates that P9-17 induced lower levels of CD86 compared to levels induced by LPS, *P* < 0.05.

**Figure 4:**
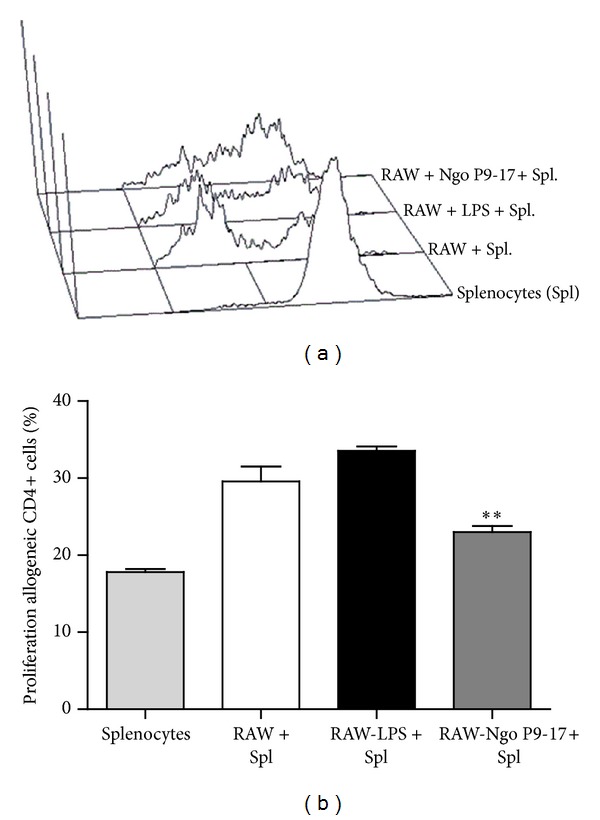
Hyporesponsive alloantigen T-cell responses induced by RAW cells infected with *N. gonorrhoeae* P9-17. Allogeneic H-2^b^ splenocytes were cultured with RAW cells treated with gonococcus, medium and LPS. At day 5, CD4+ T-cell proliferation was determined by CFSE dilution analysis. Representative histogram plots are shown in the upper panel. Percentages of proliferation are indicated in the upper left quadrant. Bars represent mean ± SEM of 5 independent experiments performed in triplicate. ** indicates that P9-17 induces lower percentages of alloantigen proliferation than LPS and other treatments *P* < 0.01.

We also sought to determine whether gonococcus changes the activation status of macrophage cell line. As expected for macrophages, the RAW 264.7 cell line responded to LPS treatment upregulating the expression of MHC class II and CD86 with 2- and 4-fold increases, respectively (Figures 3(a) and 3(b)). In contrast, P9-17 just slightly increased the expression of MHC class II, although differences did not reach statistical significance (Figure 3(a)) compared with cells in medium only. Interestingly, the gonococcal variant P9-17 did not stimulate expression of CD86 (Figure 3(b)) and levels of expression were significantly different from those reached by LPS stimulation (Figure 3(b)). Effectiveness of the immune response is conditioned by the number of MHCes: peptide that cells express (signal 1), the nature of their costimulation (signal 2), and instruction by cytokines that these cells are capable of delivering to the T lymphocyte (signal 3). Consequently, we found that gonococcal variant P9-17 treated RAW cells inhibit CD4+ T cell proliferation (Figure 4).

## 4. Discussion

Pathogenic microorganisms have developed multiple mechanisms of immune evasion to avoid eradication by the host. Several mechanisms have been described for bacterial evasion of immune response, such as prevention of opsonization, toxin secretion, disruption of mucosal barriers, modification of pattern molecules, uptake induction and phagosome escape, persistency within endosomes,interference with cytokine secretion, interference with antigen presentation, and inhibition of T- and B-cell functions, among others [27]. In this regard, *Neisseria gonorrhoeae* does not seem to be the exception, and, in particular, it is able to interfere directly with adaptive immune response, as occurs with antigenic variation [10] and phagosome subversion [28]. As well, it inhibits CD4+ T-cell proliferation, preventing adaptive immune response [19]. We recently reported that during experimental infection of the mouse, gonococcus induces an increase of regulatory T cells and infiltration of TGF-*β*1 positive cells in the uterine stroma of infected animals [17], which may also be a mechanism of immune evasion as this type of T cells induces tolerance. Other, studies in a murine model demonstrated that *N. gonorrhoeae* enhances TGF-*β*1 production and thereby promotes Th17-dependent response, with the consequent deployment of Th1/Th2 protective response [18]. Recent studies by Duncan and colleagues showed that *N. gonorrhoeae *suppresses the ability of dendritic cells (DC) to induce CD4+ T-cell proliferation and leads to upregulation of cell surface and secreted proteins with immunosuppressive properties [21].

The present report has focused on responses elicited by gonococcus on antigen-presenting cells such as *macrophages*, which trigger adaptive immunity during initial interactions with the bacteria. Here, for the first time we have demonstrated that, although gonococcus induces proinflammatory cytokines, as well as regulatory cytokines in murine macrophage cell line, a shift towards anti-inflammatory cytokine production occurs. In addition, gonococcus was highly inefficient in upregulating MHC and CD86, two of the most important molecules involved in antigen presentation and T-cell activation. Consequently, we showed that infected macrophages have weak allogeneic T-cell stimulatory activity.

In particular, gonococcus induces the secretion of the proinflammatory cytokines TNF-*α* and IL-6 in APCs. This suggests that DC and macrophages, which are usually located in the stroma of the genital tract of the mouse, are at least in part responsible for the increased levels of TNF-*α* and IL-6 observed in the vaginal secretions of Balb/c mice after gonococcal infection [29]. Mouse spleen cells or genital tract tissue explants, stimulated with *N. gonorrhoeae* secrete IL-17, IL-22, and IL-6, but not other inflammatory cytokines typical of Th1 or Th2 response [30]. However, gonococcus also induces an increase in regulatory cytokines, IL-10, and TGF-*β*1 in mouse macrophage cell line. Recently, important populations of macrophages have come to light which play a role in limiting inflammation during innate and adaptive immune response [24]. These regulatory macrophages produce high levels of IL-10 and have a potent T-cell suppressive function [31]. It is well established that IL-10 inhibits TLR-induced T-cell activation by commensal and pathogenic microorganisms and antigen presentation through the repression of inflammatory cytokine production and inhibition of expression of MHC class II and costimulatory molecules [16, 32]. Secretion of IL-10 by *N. gonorrhoeae*-exposed DC suppresses antigen-induced T-cell proliferation [21]. This mechanism is also observed in *Chlamydia trachomatis* infections [33] and commensal bacteria in other mucosal surfaces [34]. Moreover, the environments of genital tract tissues of the mouse and human likely contribute to anti-inflammatory shifting, as they express important levels of regulatory cytokines [35–37]. These data correlate well with human studies that have demonstrated that IL-10 concentration measured in endocervix and cervical mucus was higher in women infected with *N. gonorrhoeae* [38, 39]. Complementarily, TGF-*β*1 controls immune response by direct inhibition of T helper (Th1 and Th2) [40], in fact when mice are treated with anti-TGF-*β* antibody during infection with gonococcus; the duration of infection is shortened by about 4 days [41]. Therefore, because the first encounter between the bacteria and the immune system occurs in the infecting mucosa, data suggest that local induction of this cytokine profile can initiate a tolerogenic type of response that might explain asymptomatic infection, low levels of antibody production, and induction of Treg cells that has been reported to occur in Balb/c mice infected with *N. gonorrhoeae* [1, 5, 17]. 

We also found that the bacterium was unable to induce significant upregulation of the cell surface costimulatory molecule CD86 in macrophages, which further indicates the anti-inflammatory or regulatory effects of gonococcus on APCs. This suggests that, although *Neisseria gonorrhoeae* is actually phagocytosed by macrophages, the bacteria can weaken antigen-presenting functions because CD86/CD28 costimulatory pathway control of immune responses, such as antibody responses and induction of cytotoxic T-cell responses, is impaired in the absence of CD28 signaling [42]. Gonococcus is also unable to induce up-regulation of MHC class II in macrophages, which are involved in antigen capture, processing, and presentation. This implies that macrophages, which can phagocytose gonococcus [43, 44], do not have a proper antigen-presenting function. In line with this evidence, we showed that infected macrophages have weak allogeneic T-cell stimulatory activity, which strongly suggests that one of the most likely mechanisms is anergy induction, due to the lack of sufficient first and second activation signals [45].

Altogether, our results indicate that gonococcus controls the immune response at the macrophage level, inducing a tolerogenic phenotype that includes regulatory cytokines and low proliferative response, which may contribute to infection without symptoms, as occurs in women and female mice. 

## References

[1] Edwards JL, Apicella MA (2004). The molecular mechanisms used by *Neisseria gonorrhoeae* to initiate infection differ between men and women. *Clinical Microbiology Reviews*.

[2] Farley TA, Cohen DA, Elkins W (2003). Asymptomatic sexually transmitted diseases: the case for screening. *Preventive Medicine*.

[3] Handsfield HH, Lipman TO, Harnisch JP, Tronca E, Holmes KK (1974). Asymptomatic gonorrhea in men: diagnosis, natural course, prevalence and significance. *The New England Journal of Medicine*.

[4] John J, Donald WH (1978). Asymptomatic urethral gonorrhoea in men. *British Journal of Venereal Diseases*.

[5] Hedges SR, Mayo MS, Mestecky J, Hook EW, Russell MW (1999). Limited local and systemic antibody responses to *Neisseria gonorrhoeae* during uncomplicated genital infections. *Infection and Immunity*.

[6] Song W, Condron S, Mocca BT (2008). Local and humoral immune responses against primary and repeat *Neisseria gonorrhoeae* genital tract infections of 17*β*-estradiol-treated mice. *Vaccine*.

[7] Anzala AO, Simonsen JN, Kimani J (2000). Acute sexually transmitted infections increase human immunodeficiency virus type 1 plasma viremia, increase plasma type 2 cytokines, and decrease CD4 cell counts. *Journal of Infectious Diseases*.

[8] Niederkorn JY (2006). See no evil, hear no evil, do no evil: the lessons of immune privilege. *Nature Immunology*.

[9] Imarai M, Varela-Nallar L, Figueroa-Gaete C (2005). Fas ligand in the uterus of the non-pregnant mouse induces apoptosis of CD4+ T cells. *Journal of Reproductive Immunology*.

[10] van der Woude MW, Bäumler AJ (2004). Phase and antigenic variation in bacteria. *Clinical Microbiology Reviews*.

[11] Harvey HA, Swords WE, Apicella MA (2001). The mimicry of human glycolipids and glycosphingolipids by the lipooligosaccharides of pathogenic Neisseria and Haemophilus. *Journal of Autoimmunity*.

[12] Moran AP, Prendergast MM, Appelmelk BJ (1996). Molecular mimicry of host structures by bacterial lipopolysaccharides and its contribution to disease. *FEMS Immunology and Medical Microbiology*.

[13] Binker MG, Cosen-Binker LI, Terebiznik MR (2007). Arrested maturation of Neisseria-containing phagosomes in the absence of the lysosome-associated membrane proteins, LAMP-1 and LAMP-2. *Cellular Microbiology*.

[14] Plant LJ, Jonsson A-B (2006). Type IV pili of *Neisseria gonorrhoeae* influence the activation of human CD4+ T cells. *Infection and Immunity*.

[15] Levings MK, Gregori S, Tresoldi E, Cazzaniga S, Bonini C, Roncarolo MG (2005). Differentiation of Tr1 cells by immature dendritic cells requires IL-10 but not CD25+CD4+ Tr cells. *Blood*.

[16] Roncarolo MG, Gregori S, Battaglia M, Bacchetta R, Fleischhauer K, Levings MK (2006). Interleukin-10- secreting type 1 regulatory T cells in rodents and humans. *Immunological Reviews*.

[17] Imarai M, Candia E, Rodriguez-Tirado C (2008). Regulatory T cells are locally induced during intravaginal infection of mice with *Neisseria gonorrhoeae*. *Infection and Immunity*.

[18] Liu Y, Russell MW (2011). Diversion of the immune response to *Neisseria gonorrhoeae* from Th17 to Th1/Th2 by treatment with anti-transforming growth factor *β* antibody generates immunological memory and protective immunity. *MBio*.

[19] Boulton IC, Gray-Owen SD (2002). Neisserial binding to CEACAMI arrests the activation and proliferation of CD4+ T lymphocytes. *Nature Immunology*.

[20] Lee HSW, Ostrowski MA, Gray-Owen SD (2008). CEACAM1 dynamics during *Neisseria gonorrhoeae* suppression of CD4 + T lymphocyte activation. *Journal of Immunology*.

[21] Zhu W, Ventevogel MS, Knilans KJ (2012). *Neisseria gonorrhoeae* suppresses dendritic cell-induced, antigen-dependent CD4 T cell proliferation. *PLoS ONE*.

[22] Gordon S, Martinez FO (2010). Alternative activation of macrophages: mechanism and functions. *Immunity*.

[23] Biswas SK, Mantovani A (2010). Macrophage plasticity and interaction with lymphocyte subsets: cancer as a paradigm. *Nature Immunology*.

[24] Mosser DM, Edwards JP (2008). Exploring the full spectrum of macrophage activation. *Nature Reviews Immunology*.

[25] Christodoulides M, Everson JS, Liu BL (2000). Interaction of primary human endometrial cells with *Neisseria gonorrhoeae* expressing green fluorescent protein. *Molecular Microbiology*.

[26] Gómez-Duarte OG, Dehio M, Guzmán CA, Chhatwal GS, Dehio C, Meyer TF (1997). Binding of vitronectin to Opa-expressing *Neisseria gonorrhoeae* mediates invasion of HeLa cells. *Infection and Immunity*.

[27] Hornef MW, Wick MJ, Rhen M, Normark S (2002). Bacterial strategies for overcoming host innate and adaptive immune responses. *Nature Immunology*.

[28] Mosleh IM, Huber LA, Steinlein P, Pasquali C, Günther D, Meyer TF (1998). *Neisseria gonorrhoeae* porin modulates phagosome maturation. *Journal of Biological Chemistry*.

[29] Packiam M, Veit SJ, Anderson DJ, Ingalls RR, Jerse AE (2010). Mouse strain-dependent differences in susceptibility to *Neisseria gonorrhoeae* infection and induction of innate immune responses. *Infection and Immunity*.

[30] Feinen B, Jerse AE, Gaffen SL, Russell MW (2010). Critical role of Th17 responses in a murine model of *Neisseria gonorrhoeae* genital infection. *Mucosal Immunology*.

[31] Cassetta L, Cassol E, Poli G (2011). Macrophage polarization in health and disease. *The Scientific World Journal*.

[32] Li MO, Flavell RA (2008). Contextual regulation of inflammation: a duet by transforming growth factor-*β* and interleukin-10. *Immunity*.

[33] Marks E, Tam MA, Lycke NY (2010). The female lower genital tract is a privileged compartment with IL-10 producing dendritic cells and poor Th1 immunity following Chlamydia trachomatis infection. *PLoS Pathogens*.

[34] Albright CA, Sartor RB, Tonkonogy SL (2009). Endogenous antigen presenting cell-derived IL-10 inhibits T lymphocyte responses to commensal enteric bacteria. *Immunology Letters*.

[35] Taylor BN, Saavedra M, Fidel PL (2000). Local Th1/Th2 cytokine production during experimental vaginal candidiasis: Potential importance of transforming growth factor-*β*. *Medical Mycology*.

[36] Srivastava MD, Lippes J, Srivastava BIS (1996). Cytokines of the human reproductive tract. *The American Journal of Reproductive Immunology*.

[37] Nocera M, Chu TM (1995). Characterization of latent transforming growth factor-*β* from human seminal plasma. *The American Journal of Reproductive Immunology*.

[38] Geisler WM, Wang C, Tang J, Wilson CM, Crowley-Nowick PA, Kaslow RA (2008). Immunogenetic correlates of *Neisseria gonorrhoeae* infection in adolescents. *Sexually Transmitted Diseases*.

[39] Cohen CR, Plummer FA, Mugo N (1999). Increased interleukin-10 in the endocervical secretions of women with non-ulcerative sexually transmitted diseases: a mechanism for enhanced HIV-1 transmission?. *AIDS*.

[40] Li MO, Flavell RA (2008). TGF-*β*: a master of all T cell trades. *Cell*.

[41] Liu Y, Islam EA, Jarvis GA, Gray-Owen SD, Russell MW (2012). *Neisseria gonorrhoeae* selectively suppresses the development of Th1 and Th2 cells, and enhances Th17 cell responses, through TGF-*β*-dependent mechanisms. *Mucosal Immunology*.

[42] Acuto O, Michel F (2003). CD28-mediated co-stimulation: a quantitative support for TCR signalling. *Nature Reviews Immunology*.

[43] Blake M, Swanson J (1975). Studies on gonococcus infection. IX. In vitro decreased association of pilated gonococci with mouse peritoneal macrophages. *Infection and Immunity*.

[44] Cooper MD, Floyd SA (1982). In vitro kinetics of phagocytosis and intracellular killing of gonococci by peritoneal macrophages from mice deficient in complement component 5. *Infection and Immunity*.

[45] Xia C-Q, Kao KJ (2003). Suppression of interleukin-12 production through endogenously secreted interleukin-10 in activated dendritic cells: involvement of activation of extracellular signal-regulated protein kinase. *Scandinavian Journal of Immunology*.

